# Parent and Provider Differences in Ratings of Mental Health and Neurodevelopmental Concerns in Children with Neurologic Disorders

**DOI:** 10.1007/s10880-023-09990-0

**Published:** 2024-02-24

**Authors:** Jessica M. Schwartzman, Zachary J. Williams, Andrew E. Molnar

**Affiliations:** 1 Department of Pediatrics, Keck School of Medicine, University of Southern California, Los Angeles, CA USA; 2 Division of Developmental-Behavioral Pediatrics, Children’s Hospital Los Angeles, 3250 Wilshire Blvd, Los Angeles, CA 90010 USA; 3 Department of Psychiatry and Behavioral Sciences, Vanderbilt University Medical Center, 1500 21st Avenue South, Suite 2200, Nashville, TN 37212 USA; 4 Medical Scientist Training Program, Vanderbilt University School of Medicine, 2209 Garland Avenue, Nashville, TN 37240 USA; 5 Department of Hearing and Speech Sciences, Vanderbilt University Medical Center, Nashville, TN USA; 6 Vanderbilt Kennedy Center, Vanderbilt University Medical Center, Nashville, TN USA; 7 Frist Center for Autism and Innovation, Vanderbilt University, Nashville, TN USA; 8 Vanderbilt Brain Institute, Vanderbilt University, Nashville, TN USA

**Keywords:** Neurologic disorders, Pediatric neuropsychology, Child psychiatry, Mental health, Neurodevelopmental concerns

## Abstract

Children with neurologic disorders face increased risks for mental health and neurodevelopmental conditions, with information often limited to parent report. To better understand mental health and neurodevelopmental needs in this population, a retrospective chart review of a convenience sample of children with neurologic disorders referred for a neuropsychological evaluation was conducted in the present study to explore interrater agreement between care team members (referring providers, parents, pediatric neuropsychologist). Qualitative and quantitative data were collected from the evaluation reports of 129 youth (9:0–17:11 years old; 51.2% of female sex) with neurologic disorders (i.e., 38.0% traumatic brain injury, 27.1% epilepsy, 14.7% premature birth, 7.8% pediatric cancer, 3.9% prenatal substance exposure, and 14.7% other) who completed an evaluation in 2019. Over half the youth were flagged for unmet neurodevelopmental and mental health concerns and analyses revealed low interrater agreement for mental health concerns (κ = .324), better agreement for neurodevelopmental concerns (κ = .511), and low sensitivity of referring providers (*Se* = .326) and parents (*Se* = .366). One-way analyses of variance uncovered important factors (e.g., symptom severity, adaptive skills) that may account for missed concerns. Findings guide recommendations to strengthen methods for understanding mental health and/or neurodevelopmental concerns in children with neurologic disorders.

**Supplementary Information:**

The online version contains supplementary material available at 10.1007/s10880-023-09990-0.

## Introduction

Children with neurologic disorders (e.g., traumatic brain injury, epilepsy, etc.) are a growing population in the United States and worldwide who are at increased risk for adverse mental health outcomes (e.g., anxiety, depression; Ferro & Boyle, 2015; Stephenson et al., 2015) and many are diagnosed with neurodevelopmental conditions (e.g., ADHD, autism; Nylander et al., 2015). If undetected, mental health and/or neurodevelopmental conditions can contribute to diminished quality of life (LaGrant et al., 2020). Furthermore, untreated concerns hinder adherence to medical regimens and the likelihood of optimal control over neurologic disorders (Blackman et al., 2011). Early screening and intervention are associated with improved long-term outcomes (e.g., quality of life, academic achievement; Kuhlthau et al., 2011). Though many factors contribute to unaddressed and untreated concerns in pediatric populations, low agreement between providers (e.g., neurologists, psychologists, nurse practitioners) and between providers-parents on these concerns can delay access to assessment and interventions (Schwartz et al., 2018). Examining provider-provider and provider-parent perspectives on mental health and/or neurodevelopmental concerns specifically in children with neurologic disorders is an important extension of this work.

Efforts to understand mental health and neurodevelopmental concerns in children with neurologic disorders have increased in recent years and contributed to improvements in personal and family well-being (Whitney et al., 2019). Still, screening and intervention standards appear differential across settings with screening processes being variable and conducted by a range of healthcare professionals and practices (Beers et al., 2017; Brown & Wissow, 2010; Wissow et al., 2013). While mental health and neurodevelopmental conditions in children with neurologic disorders are appreciated in the growing literature, research on interrater agreement between providers and providers-parents (i.e., primary care team members) is largely limited. Though many parents lack formal training in child development and are therefore, more likely to be discrepant from professional opinions, their perspectives afford valuable insight to the child’s daily life (i.e., outside of a clinical visit with a provider). Furthermore, parent perspectives are important in conceptualizing a child given a parent’s role in accessing youth medical and psychiatric services, which predicate on a parent recognizing the needs of the child and responding accordingly (Burnett-Zeigler & Lyons, 2010; Chan et al., 2023).

Several barriers have been identified in efforts to address mental health and neurodevelopmental needs among children with neurologic disorders including little research on the reliability and validity of screening tools in this population (Bennett et al., 2019) and a primary emphasis on treating medical concerns (Vinall et al., 2016). In addition, interrater disagreement between youth, their parents, and care providers may be common with previous work reporting differences in ratings of youth quality of life (Eiser & Varni, 2013a; Pinquart & Shen, 2011; Vetter et al., 2012), which were associated with later access to intervention (de Los Reyes, 2011). Investigators emphasized the importance of understanding both the direction and magnitude of informant discrepancies (Eiser & Varni, 2013a), which may inform predictions of patient outcomes and family adherence to treatment regimens (de Los Reyes, 2011). However, to our knowledge, no studies have examined the direction and magnitude of interrater agreement between care team providers (e.g., neuropsychologist, neurologist) and providers-parents on mental health and neurodevelopmental concerns in children specifically with neurologic disorders.

While parents and referring providers contribute important perspectives on youth, neuropsychological evaluations result in a more thorough phenotyping of the child across many domains (e.g., cognition, language, medical history, etc.) and opportunities for reconciling multiple provider and parent perspectives. Typically in evaluations, neuropsychologists conduct a meticulous review of a patient’s medical records to obtain histories and observations from multidisciplinary providers (e.g., primary care provider, neurologist, psychiatrist), as well as referral information. With this knowledge, neuropsychologists interview parents to obtain additional information (e.g., developmental history, academic performance, etc.), with opportunities to clarify discrepancies or points of confusion. While neuropsychological evaluations largely focus on assessing cognitive functions in individuals with neurologic conditions, there also exist opportunities to evaluate mental health status. Through this process, neuropsychologists balance perspectives from multidisciplinary providers and parents, in combination with neuropsychological tests and behavioral observations, to formulate an extensive conceptualization of the patient in a neuropsychological report. Therefore, the rich phenotyping available in these reports may be an important method to understand mental health and/or neurodevelopmental concerns in children with neurologic disorders from diverse perspectives.

### Present Study

In the present study, a retrospective chart review of a convenience sample of children with neurologic disorders referred for a neuropsychological evaluation was conducted to explore interrater agreement between care team members (i.e., referring providers, parents, pediatric neuropsychologist) in detecting mental health and/or neurodevelopmental concerns in these children. A primary aim was to examine the direction and magnitude of interrater agreement between multiple care team members in detecting mental health and/or neurodevelopmental concerns. We also examined the sensitivity and specificity of referring providers and parents in identifying concerns when comparing their ratings to those of the pediatric neuropsychologist. Exploratory analyses investigated potential factors (e.g., age, intellectual abilities, adaptive skills) that may contribute to mental health and/or neurodevelopmental concerns in this sample. We hypothesized that interrater agreement would be low between referring providers, parents, and the pediatric neuropsychologist on youth mental health and neurodevelopmental concerns and that certain youth factors (e.g., age, intellectual abilities, adaptive behaviors) would be associated with more severe mental health concerns.

## Materials and Methods

### Participants

The sample included children and adolescents with neurologic disorders (e.g., traumatic brain injury, epilepsy, etc.) without intellectual disability who were referred for a neuropsychological evaluation with a licensed pediatric neuropsychologist (author AEM) in an outpatient clinic in 2019. For the study, inclusion criteria included participants: (a) 9:0–17:11 years old seen in 2019, (b) English speaking, (c) with a primary neurological condition (e.g., traumatic brain injury, epilepsy, etc.), (d) and with a full-scale IQ (FSIQ) of 70 or above on the Wechsler Abbreviated Scale of Intelligence, Second Edition (Wechsler & Hsiao-pin, 2011) or Wechsler Intelligence Scale for Children, Fifth Edition in order to complete self-report measures as validated (Cormier et al., 2016).

Exclusion criteria included patients: (a) younger than nine years old due to the age limits of self-report questionnaires, (b) without a neurologic disorder, or (c) with FSIQ < 70. Informed consent for a neuropsychological evaluation was collected from parents prior to each appointment. The present study was a retrospective analysis of de-identified information, and all study procedures were approved by the Institutional Review Board at Vanderbilt University (#200598).

### Procedures

#### Neuropsychological Evaluation

For all neuropsychological evaluations, a standard template was used to first collect phenotyping data on patients and then to write the neuropsychological report, including: (a) demographic information, (b) referring provider notes and referral reason(s), (c) review and summary of histories from medical records, (d) intake interview with parents/caregivers, including routine questions regarding existing mental health and/or neurodevelopmental concerns or diagnoses, (e) behavioral observations, (f) diagnostic impressions formulated by the neuropsychologist using the collected information, (g) intervention recommendations (e.g., educational, psychological, medical), and (h) raw and standardized scores from neuropsychological tests and psychological questionnaires. The structure of the evaluations and reports was consistent across participants (see “Measures” section).

#### Data Collection

The primary author (JMS), a licensed clinical psychologist, read through all of the reports to code the data summarized in each report (see details below); discrepancies in codes were resolved with the pediatric neuropsychologist (last author AEM). Demographic information (i.e., child age and sex) was collected from each report and referral information including: (a) type of referring provider (e.g., neurologist, nurse practitioner, etc.), (b) referral reason (e.g., cognitive concerns, learning difficulties, etc.), and (c) medical diagnosis (e.g., traumatic brain injury, epilepsy, etc.).

To investigate reliability between raters (i.e., referring provider, parent/caregiver, pediatric neuropsychologist), the presence/absence of mental health concerns raised by any of the three raters were coded as dichotomous variables (i.e., present vs. absent). The “presence” of mental health concerns was coded as “1” and operationally defined as any reference to mental health symptoms (e.g., “she worries a lot”, “his mood is irritable”) or established diagnoses (e.g., generalized anxiety disorder, posttraumatic stress disorder) by a given rater. While certain child experiences described by raters (e.g., “she worries a lot,” or, “his mood is irritable”) may be common and/or developmentally appropriate, only statements that were described as ongoing difficulties for a given child, uncommon for the child’s developmental period, and/or causing distress were categorized as mental health symptoms. If mental health concerns were reported (i.e., coded as “1”), then the type (e.g., anxiety symptoms = 1; depression symptoms = 2; etc.) and severity of concerns were nominally coded (i.e., established diagnosis = 1; subthreshold symptoms = 2). The “absence” of mental health concerns (i.e., coded as “0”) was operationally defined as the lack of reference to any mental health symptoms or established diagnoses from a rater.

The same coding scheme was applied to neurodevelopmental concerns in the same format of dichotomous variables (i.e., presence = 1; absence = 0). The “presence” of neurodevelopmental concerns was coded as “1” and operationally defined as any reference to neurodevelopmental traits (e.g., “difficulty paying attention,” or, “social challenges”) or established diagnoses (e.g., ADHD, Autism Spectrum Disorder). Certain child experiences (e.g., “difficulty paying attention,” or, “social challenges”) were classified as neurodevelopmental concerns if they were described as ongoing difficulties for a given child and/or causing distress.

Lastly, first-time mental health and/or neurodevelopmental diagnoses were coded as dichotomous variables (i.e., first-time mental health diagnosis = 1; existing mental health diagnosis = 0).

### Measures

Several self- and parent-rated questionaries and neuropsychological assessments were administered to participants as part of the standard evaluation and used by the neuropsychologist to formulate diagnoses and recommendations in the report. The Multidimensional Anxiety Scale for Children, Second Edition (MASC-2; March et al., 1997) was completed by youth to assess emotional, physical, cognitive, and behavioral symptoms of anxiety. The Children’s Depression Inventory, Second Edition (CDI-2; Kovacs, 2015) was completed by youth to assess cognitive, affective, and behavioral aspects of mood and depressive symptoms. Youth and parents completed the Conners, Third Edition, Parent and Self-Report Forms (Conners-3; Conners, 2008) to assess cognitive, behavioral, and emotional problems among youth, with a focus on ADHD and comorbid disorders. Youth with T-scores ≥ 60 on the MASC-2, CDI-2, and Conners-3 surpassed clinical thresholds.

Additionally, the WASI-II (Wechsler, 2011) or WISC-V (Cormier et al., 2016) were administered to assess intellectual functioning. The Full Scale IQ-4 Subtests (FSIQ-4) of the WASI-II and FSIQ of the WISC-V were coded as a single variable as they are correlated (Raiford et al., 2016; Zhou & Raiford 2011). Lastly, parents completed the Adaptive Behavior Assessment, Third Edition (ABAS-3; Harrison & Oakland, 2018) to assess youth adaptive skills. The ABAS-3 includes a General Adaptive Composite, which is comprised of three domains: practical, social, and conceptual abilities. Raw total and domain scores are converted to standard scores.

### Statistical Analyses

#### Detection of Concerns and Inter-rater Agreement

To test agreement between raters (referring provider, parent, neuropsychologist) about the presence or absence of mental health and/or neurodevelopmental concerns, we examined three-way inter-rater agreement using Fleiss’s kappa coefficient (Fleiss, 1971). In addition, agreement between each pair of raters (i.e., referring provider vs. parent, parent vs. neuropsychologist, referring provider vs. neuropsychologist) was quantified using Cohen’s kappa (Cohen, 1960). These ratings were computed twice: (1) with all of the neuropsychologist’s findings (i.e., both DSM-5 diagnoses and subthreshold symptoms) counted as “concerns” and, (2) with only DSM-5 diagnoses counted as “concerns.”

To assess whether the differences between pairs of kappa coefficients were statistically significant, we utilized permutation testing (10,000 permutations) based on the method of McKenzie and colleagues (1997). All statistical tests were performed in the R statistical computing environment (R Core Team, 2020). In addition, we calculated the sensitivity (*Se*) and specificity (*Sp*) of referring provider and parent concerns (along with 95% bootstrapped confidence intervals) when predicting a finding of a mental health or neurodevelopmental concern by the neuropsychologist.

#### Comparison of Subgroups

To explore potential factors contributing to missed concerns, we examined whether patients with undiagnosed mental health or neurodevelopmental conditions differed from others on a range of clinical and demographic variables. Children in our sample were divided into three subgroups based on mental health concerns: (1) MH-P_0_N_0_: no mental health concerns from either the parent (P_0_) or neuropsychologist (N_0_; *n* = 31; 24.03% of sample), (2) MH-P_0_N_1_: no mental health concerns reported by the parent (P_0_) but diagnosed with a mental health condition by the neuropsychologist (N_1_; *n* = 52; 40.31% of sample), and (3) MH-P_1_N_1_: mental health concerns reported by the parent (P_1_) and diagnosed with a mental health condition by the neuropsychologist (N_1_; *n* = 46; 35.66% of sample). Analogous groups were also formed based on parental concerns and diagnosis of a neurodevelopmental disorder by the neuropsychologist (ND-P_0_N_0_: *n* = 40; ND-P_0_N_1_: *n* = 44; ND-P_1_N_1_; *n* = 45).

A number of demographic and clinical variables were compared between the groups using one-way analysis of variance (ANOVA), with omega-squared (*ω*^*2*^) as the index of effect size (Okada, 2013). Significant results were followed up by post-hoc tests using Tukey’s honest significant difference test. Analyses were conducted twice, once when dividing children based on mental health outcomes and a second time when dividing children based on neurodevelopmental outcomes. Variables of interest included chronological age, sex, FSIQ, adaptive behavior scores (ABAS-3), anxiety (MASC-2), depression (CDI-2), ADHD symptoms (Conners-3), and other indices from the Conners-3 including disruptive behaviors, ADHD-related cognitive complaints, and social functioning.

## Results

### Participants and Reported Concerns

A total of 129 children and adolescents (62 males, *M* ± *SD* age = 13.21 ± 2.88 years) were included in the study and referral reasons included either cognitive concerns (59.2%) or academic/learning difficulties (40.8%; see Table 1). No participants were referred for an evaluation due to primary mental health concerns. Neurologists were the most common type of referring provider (51.1%) and other types of providers included primary care physicians (28.7%), nurse practitioners (12.4%), and psychiatrists (7.8%).

**Table 1:** Demographics and clinical characteristics of sample

Characteristic	Full sample (*N* = 129)
Age (years)	13.21 (2.88)
Female (sex)	67 (51.2%)
Neurologic disorder	
Traumatic brain injury	49 (38.0%)
Epilepsy	35 (27.1%)
Premature birth	19 (14.7%)
Pediatric cancer	10 (7.8%)
Prenatal substance exposure	5 (3.9%)
Other diagnosis^a^	19 (14.7%)
Services at time of evaluation	
Individualized education plan	32 (24.8%)
504 Plan	20 (15.5%)
Pull-out educational services	3 (2.3%)
Behavior therapy for mental health disorder	17 (10.1%)
Behavior therapy for neurodevelopmental needs	33 (25.6%)
FSIQ (standard score)	91.76 (12.14)
ABAS-3 GAC (standard score)	85.49 (15.42)
Mental health symptoms and neurodevelopmental traits	
MASC-2 total T-score (self)	58.71 (13.42)
CDI-2 total T-score (self)	58.66 (12.95)
Conners-3 inattention T-score (self)	66.36 (15.28)
Conners-3 inattention T-score (parent)	71.27 (15.98)
Conners-3 hyperactivity T-score (self)	60.91 (15.39)
Conners-3 hyperactivity T-score (parent)	65.91 (17.13)

Continuous variables are presented as *M* (SD), whereas categorical variables are presented as *n* (%). More than one chronic health condition could be reported by a given child, and thus percentages do not total to 100%

*FSIQ* full-scale intelligence quotient; *ABAS-3* Adaptive Behavior Assessment System-3; *GAC* general adaptive composite; MASC-2 Multidimensional Anxiety Scale for Children-2; *CDI-2* Children’s Depression Inventory-2

^a^Other diagnoses included conditions such as cerebral palsy, spina bifida, tuberous sclerosis, encephalopathy, chronic migraine, multiple sclerosis, and pediatric autoimmune neuropsychiatric syndrome, among others

Rates of mental health and neurodevelopmental concerns varied substantially between the three raters, with the pediatric neuropsychologist noting a larger number of cases than both parents and referring providers (see Table 2). Only one child with an existing mental health diagnosis (anxiety) and one child with an existing neurodevelopmental diagnosis (ADHD) were judged by the neuropsychologist to no longer meet criteria for those conditions.

**Table 2:** Diagnosis frequencies and interrater agreement for mental health and neurodevelopmental concerns

Informant	Mental health concerns		Neurodevelopmental concerns	
	New Dx	Previous Dx	New Dx	Previous Dx
Referring provider	11 (8.5%)	23 (17.8%)	6 (4.7%)	38 (29.5%)
Parent	20 (15.5%)	27 (20.9%)	8 (6.2%)	38 (29.5%)
Neuropsychologist (DSM-5 Dx)	23 (17.8%)	11 (8.5%)	13 (10.1%)	37 (28.7%)
Neuropsychologist (subthreshold symptoms/traits)	49 (38.0%)	15 (11.6%)	39 (30.2%)	0 (0.0%)

Informant combination	Agreement (%)	Agreement (κ)	Agreement (%)	Agreement (κ)
All informants				
Neuropsychologist: DSM-5 + subthreshold	66.4	0.324 [0.208, 0.458]	75.7	0.511 [0.389, 0.618]
Neuropsychologist: DSM-5 only	81.4	0.555 [0.430, 0.668]	95.3	0.899 [0.818, 0.947]
Referring provider/parent	89.9	0.769 [0.637, 0.874]	98.4	0.966 [0.880, 1.0]
Referring provider/neuropsychologist				
Neuropsychologist: DSM-5 + subthreshold	50.4	0.203 [0.125, 0.295]	63.6	0.350 [0.235, 0.469]
Neuropsychologist: DSM-5 only	78.3	0.443 [0.262, 0.612]	93.8	0.866 [0.749, 0.934]
Parent/neuropsychologist				
Neuropsychologist: DSM-5 + subthreshold	58.9	0.280 [0.178, 0.399]	65.1	0.371 [0.251, 0.494]
Neuropsychologist: DSM-5 only	76.0	0.449 [0.280, 0.597]	93.8	0.867 [0.749, 0.935]

Informant Combination	Sensitivity	Specificity	Sensitivity	Specificity
Referring provider/neuropsychologist				
Neuropsychologist: DSM-5 + subthreshold	0.326 [0.232, 0.421]	1.0 [1.0, 1.0]	0.459 [0.353, 0.565]	0.977 [0.932, 1.0]
Neuropsychologist: DSM-5 Only	0.853 [0.779, 0.916]	0.588 [0.412, 0.765]	0.918 [0.859, 0.965]	0.977 [0.932, 1.0]
Parent/neuropsychologist				
Neuropsychologist: DSM-5 + subthreshold	0.366 [0.268, 0.476]	0.979 [0.936, 1.0]	0.470 [0.361, 0.578]	0.978 [0.935, 1.0]
Neuropsychologist: DSM-5 only	0.890 [0.817, 0.951]	0.532 [0.383, 0.681]	0.928 [0.867, 0.976]	0.957 [0.891, 1.0]

Diagnosis frequencies for each informant are presented as *N* (%). Kappa, sensitivity, and specificity coefficients are accompanied by 95% bootstrapped confidence intervals. “DSM-5 + Subthreshold” indicates that “positive” neuropsychologist findings include both disorders meeting full DSM-5 diagnostic criteria and subthreshold symptoms, where “DSM-5 Only” indicates that only the former are counted as “positive” findings

*Dx*  diagnosis

### Inter-rater Agreement

#### Mental Health Concerns

Three-way agreement between the raters regarding mental health concerns was poor overall (κ = .324), with agreement increasing when the neuropsychologist’s findings were restricted to only DSM-5 diagnoses (κ = .555; see Table 2). We found excellent agreement between referring providers and parents (κ = .769), but less agreement between the neuropsychologist (including DSM-5 disorders and subthreshold symptoms) and both the referring providers (κ = .203) and parents (κ = .280). Permutation tests demonstrated that neuropsychologist (_N_) and referring provider (_RP_) agreement (κ_N,RP_), and neuropsychologist (_N_) and parent (_P_) agreement (κ_N,P_), were significantly smaller than agreement between parents and referring providers (κ_P,RP_; *p*s < .001). However, κ_N,P_ and κ_N,RP_ did not differ significantly from one another (*p* = .164). When “positive” neuropsychologist findings were restricted to only include DSM-5 diagnoses, pairwise agreement increased significantly between the neuropsychologist and both other informants (see Table 2 for details), with no significant difference between κ_N,P_ and κ_N,RP_ (*p* = .918). However, in both of these cases, agreement was still significantly poorer than agreement between parents and referring providers (*p*s < .002).

#### Neurodevelopmental Concerns

Three-way agreement between raters regarding neurodevelopmental concerns was higher than that for mental health concerns (see Table 2). Agreement between the three raters increased when considering only DSM-5 neurodevelopmental diagnoses (κ = .899), as compared to the agreement when considering both DSM-5 diagnoses and subthreshold symptoms (κ = .511). There was near perfect agreement between referring providers and parents (κ = .966), but less agreement between neuropsychologist (including DSM-5 disorders and subthreshold symptoms) and both the referring providers (κ = .350) and parents (κ = .371). Permutation tests again demonstrated that κ_N,RP_ and κ_N,P_ were significantly smaller than κ_P,RP_ (*p*s < .001), although κ_N,P_ and κ_N,RP_ did not differ significantly from one another (*p* = .494). After restricting “positive” neuropsychologist findings to only those containing DSM-5 neurodevelopmental disorder diagnoses, however, pairwise kappa coefficients demonstrated greater agreement between the neuropsychologist and both other informants, with minimal difference between κ_N,RP_ and κ_N,P_ (*p* = .722).

### Clinical Comparison of Subgroups

#### Mental Health Concern Subgroups

The three subgroups based on mental health concerns (i.e., no concerns from parent or neuropsychologist, MH-P_0_N_0_; no concerns reported by parent, but endorsed by neuropsychologist, MH-P_0_N_1_; concerns reported by parent and neuropsychologist, MH-P_1_N_1_) did not significantly differ in terms of age (*F*(2,126) = .092, *p* = .912, *ω*^*2*^ = −.014). The three subgroups did not differ by sex ratio (*X*^*2*^(2) = 1.49, *p* = .475, *V* = .107) nor FSIQ (*F*(2,126) = .280, *p* = .757, *ω*^*2*^ = −.011). Estimated marginal means and confidence limits for all group comparisons are displayed in Supplemental Table 1. The three groups did not differ on measures of adaptive behavior. However, large group differences were seen in youth self-rated anxiety with the MH-P_0_N_0_ group reporting significantly lower anxiety scores than both the MH-P_0_N_1_ and MH-P_1_N_1_ groups. However, there were no significant differences in youth self-rated anxiety between the MH-P_0_N_1_ and MH-P_1_N_1_ groups. A similar pattern of results was noted for youth self-reported depression symptoms, with lower scores in the MH-N_0_ group compared to the MH-P_0_N_1_ and MH-P_1_N_1_ groups, but no significant difference between the MH-P_0_N_1_ and MH-P_1_N_1_ groups.

Significant group differences were also observed on all subscales of the self- and parent-reported Conners-3, except for hyperactivity, learning problems, and peer relations (see Supplemental Table 1). Notably, for all self- and parent-reported Conners subscales, pairwise group differences between MH-P_0_N_1_ and MH-P_1_N_1_ groups did not reach statistical significance (all *p*s > .149).

#### Neurodevelopmental Concern Subgroups

The three subgroups based on neurodevelopmental concerns (no concerns from parent or neuropsychologist, ND-P_0_N_0_; no concerns reported by parent, but endorsed by neuropsychologist, ND-P_0_N_1_; concerns reported by parent and neuropsychologist, ND-P_1_N_1_) did not significantly differ in terms of age nor FSIQ. However, they did significantly differ in terms of sex ratio (*X*^*2*^(2) = 7.75, *p* = .021, *V* = .245), with a female predominance in the ND-P_0_N_0_ and ND-P_0_N_1_ groups and a male predominance in the ND-P_1_N_1_ group. Estimated marginal means and confidence limits for all mental health group comparisons are displayed in Supplemental Table 2. Unlike the mental-health groups, neurodevelopmental groups differed substantially in all adaptive behavior scores on all ABAS-3 subscales and the general adaptive composite. However, in these comparisons, group differences were entirely driven by the ND-P_1_N_1_ group, which exhibited significantly lower scores than both the ND-P_0_N_0_ and ND-P_0_N_1_ groups. In contrast, all pairwise ABAS-3 score comparisons between MH-N_0_ and MH-P_0_N_1_ groups did not reach statistical significance (all *p*s > .182).

The neurodevelopmental subgroups did not significantly differ in youth self-reported symptoms of anxiety or depression, although large and significant differences were found in all self- and parent-reported Conners-3 subscales. Notably, while all Conners-3 group contrasts were driven in part by significantly higher scores in the ND-P_1_N_1_ group (and often the ND-P_0_N_1_ group as well) compared to the ND-P_0_N_0_ group, significant differences were found between the ND-P_0_N_1_ and ND-P_1_N_1_ groups in terms of: youth self-reported hyperactivity and defiance/aggression and parent-reported inattention, hyperactivity, learning problems, defiance/aggression, executive functioning, and peer relations. No between-group comparisons based on neurodevelopmental concern groups were significantly altered by the addition of sex to the models, and thus we chose to only present the results from one-way ANOVAs.

## Discussion

The current study examined inter-rater agreement between three care team members (referring providers, parents, pediatric neuropsychologist) serving children with neurologic disorders and showed divergent perspectives; the neuropsychologist identified mental health and neurodevelopmental concerns for the first time in many children. Findings mirror previous research identifying differences in ratings of health-related quality of life across raters (Eiser & Varni, 2013b; Pinquart & Shen, 2011) and contribute to the literature by comparing ratings of mental health and/or neurodevelopmental concerns in children with neurologic disorders across these raters. It is important to note that a significant portion of the sample exhibited undiagnosed mental health and/or neurodevelopmental concerns prior to the neuropsychological evaluation that were not reported by parents or referring providers and instead, referral reasons focused on cognitive and/or academic difficulties.

Ratings of DSM-5 mental health diagnoses and subthreshold symptoms revealed in this study suggest that children with neurologic disorders are at risk for adverse mental health outcomes and/or neurodevelopmental conditions, many of which may not be reported by referring providers or parents for various reasons. Our findings add to the growing literature emphasizing the importance of identifying and communicating mental health concerns in this high risk population (Pinquart & Shen, 2011b). Perspectives from parents, referring providers, and neuropsychologists, among other team members, are equally important in understanding areas of strength and difficulty for children with neurologic disorders. Unsurprisingly, findings suggest that direct assessment (e.g., mental health questionnaires, neuropsychological evaluation, etc.) of psychiatric symptoms may be optimal to early screening and intervention efforts. Though different perspectives are common, low agreement between raters may interfere with clear diagnostic conceptualizations and/or treatment plans. Without access to effective interventions, youth who are advancing in age may have persisting or worsening mental health and/or neurodevelopment needs (Kuhlthau et al., 2011; Novins et al., 2013). This applies to both ongoing cognitive deficits that may be unrecognized and yield more learning challenges across schooling, and also persisting mental health symptoms including trauma, other anxiety presentations, or even depression.

﻿The present study was limited to a retrospective chart review with a small sample and thus, findings should be interpreted with caution as medical records may be limited in flagging all concern areas for a patient. Referring providers were not interviewed in this study so it is possible that providers were already cognizant of mental health and/or neurodevelopmental concerns in youth. Alternatively, it is possible that referring providers were aware of child mental health and/or neurodevelopmental concerns and directing families to other services (e.g., psychotherapy, psychiatrist) to address these needs. Furthermore, with a small convenience sample of children with diverse neurologic disorders, it is not feasible to draw definitive conclusions about mental health and/or neurodevelopmental concerns in this population. It is also possible that differences in mental health and/or neurodevelopmental concerns may be attributed to different neurologic conditions; for example, children born prematurely may have different needs than children with a traumatic brain injury. Future studies with larger subgroups of children with neurologic conditions are needed to tease apart these potential differences.

The pediatric neuropsychologist employed multimethod (e.g., interview, questionnaires, neuropsychological assessments), multi-informant (e.g., parent, youth) methods, which likely contributed to increased detection of mental health concerns for the first time in this sample. In addition, the neuropsychologist deliberately and routinely asked each youth about mood, symptoms of anxiety, experiences of depression, social relationships, and exposure to traumatic experiences, *regardless of primary referral reason*. It is evident that these multimethod, multi-informant assessments significantly increased the likelihood that youth at-risk for psychiatric disorders were detected accurately, even within one neuropsychological evaluation. Findings may highlight the importance of expanding the traditional scope of neuropsychological evaluations (i.e., focus on neurocognitive domains and functioning) to routinely assessing for mental health and neurodevelopmental symptoms during evaluations.

In contrast, inter-rater agreement was higher between the three raters in ratings of DSM-5 neurodevelopmental diagnoses and subthreshold symptoms than agreement for mental health concerns. Higher agreement may be explained by near perfect agreement between referring providers and parents; however, neurodevelopmental concerns may not be fully captured by these two raters given that the neuropsychologist identified 13 children with neurodevelopmental concerns above the DSM-5 diagnostic threshold and 39 additional children with subthreshold symptoms for the first time. Findings may be explained, in part, by increased awareness of risks for inattention, hyperactivity, social difficulties, or other neurodevelopmental concerns in children with neurologic disorders among providers and parents given extensive research and practice guidelines in this area (Blackman et al., 2011; Maslow et al., 2011). Our findings align with those in the broader pediatrics literature that indicate poor inter-rater agreement between different care team members (e.g., parents, teachers, providers, etc.) on mental health (Brown & Wissow, 2010; Wissow et al., 2013) and neurodevelopmental (Wolraich et al., 2004) concerns in youth.

Findings from the three subgroups of mental health concerns (MH-P_0_N_0,_ MH-P_0_N_1_, MH-P_1_N_1_) revealed significant differences across subgroups for certain domains of well-being (e.g., anxiety, depression, attention, executive function), but not others (e.g., adaptive behaviors). For both youth self-rated anxiety and depression, the two subgroups with mental health concerns (i.e., MH-P_0_N_1_, MH-P_1_N_1_) exhibited significantly higher internalizing symptoms (1.5–2 SD) than children without mental health concerns (i.e., MH-P_0_N_0_); however, differences between the two subgroups with mental health concerns were not significant. Given this, it appears that symptom severity may not entirely explain why some parents report mental health concerns that match those of the neuropsychologist while other parents do not. In addition to elevated anxiety and depression scores, youth in the two mental health subgroups (i.e., MH-P_0_N_1_, MH-P_1_N_1_) also endorsed inattention, hyperactivity/impulsivity, and learning problems above the clinical cutoffs, which may suggest an additive challenge to ongoing mental health concerns. Therefore, these five domains of functioning—anxiety, depression, inattention, hyperactivity/impulsivity, and learning problems—may be particularly important for providers, parents, and other care team members to regularly assess, monitor, and address during routine follow-up neurological care.

Children were also categorized into subgroups based on presence of neurodevelopmental concerns and findings revealed more robust patterns of differences between subgroups for certain domains (e.g., adaptive behaviors, attention, executive function), but not indices of mental health (e.g., anxiety, depression). Findings revealed significant differences between the three neurodevelopmental subgroups with the highest symptom severity among those with concerns noted by parents and the neuropsychologist (i.e., ND-P_1_N_1_) for adaptive behaviors on the ABAS-3 and all subscales of the parent-rated Conners-3. It is possible that reduced adaptive behaviors and high symptom severity are important factors in understanding why some parents report neurodevelopmental concerns and other parents do not.

### Limitations

There are limitations to the present study that warrant a discussion. First, the present study was a retrospective chart review of a convenience sample of children with neurologic disorders and thus, is not fully representative of this population. Second, the sample included youth with diverse neurologic disorders with heterogenous etiologies and treatment plans, which limit findings to the group as a whole rather than specific medical conditions. Third, questionnaires (e.g., MASC-2, CDI-2) were not systematically completed by youth and the three raters, which limits findings and highlights an important future direction. Fourth, additional demographic factors (e.g., race, ethnicity, socioeconomic status) were not available and hinder our ability to understand how these factors may influence mental health and/or neurodevelopmental concerns in this population. Lastly, detailed information about pre-existing mental health and/or neurodevelopmental disorders (e.g., age of diagnosis, diagnosing provider) were not available.

## Conclusion

Findings from the present study revealed high rates of undiagnosed mental health and/or neurodevelopmental concerns in a sample of children with diverse neurologic disorders. In particular, anxiety, depression, inattention, hyperactivity/impulsivity, and learning problems appear to be prominent challenges for children with neurologic disorders that place them at elevated risk for psychiatric disorders. Collectively, findings support the use of multimethod, multi-informant measures to comprehensively assess mental health and neurodevelopmental concerns in children with neurologic disorders.

### Supplementary Information

Below is the link to the electronic supplementary material.

Supplementary file1 (DOCX 23 KB)

Supplementary file2 (DOCX 21 KB)

## Data Availability

Available from corresponding author upon request and with data use agreement.
