# Supplementary material for: Parent and Provider Differences in Ratings of Mental Health and Neurodevelopmental Concerns in Children with Neurologic Disorders
Source: J Clin Psychol Med Settings. 2024 Feb 24;31(3):526–36. doi: 10.1007/s10880-023-09990-0 (PMC11333177; doi:10.1007/s10880-023-09990-0)
Supplement: Supplementary file 1 — Supplementary file1 (DOCX 23 KB) [file 10880_2023_9990_MOESM1_ESM.docx]

**Supplemental Table 1.**

***Estimated Marginal Means and Comparisons Among Mental Health Concern Groups***

| **Outcome** | **MH-P_0_N_0_ Group (EMM [95% CI])** | **MH-P_0_N_1_ Group (EMM [95% CI])** | **MH-P_1_N_1_ Group (EMM [95% CI])** | **Omnibus Test** | ***p*** | **ω^2^** | **Significant Contrasts** |
| --- | --- | --- | --- | --- | --- | --- | --- |
| Adaptive Behavior (Standard Score) |  |  |  |  |  |  |  |
| ABAS-3 GAC | 89.9 [84.0, 95.7] | 85.0 [80.4, 89.5] | 83.1 [78.2, 87.9] | *F*(2,108) = 1.60 | 0.206 | 0.011 | — |
| ABAS-3 Conceptual | 87.8 [81.6, 93.9] | 85.2 [80.3, 90.1] | 83.3 [78.1, 88.4] | *F*(2,110) = 0.61 | 0.546 | -0.007 | — |
| ABAS-3 Social | 93.0 [87.0, 99.0] | 87.6 [83.0, 92.3] | 85.2 [80.1, 90.2] | *F*(2,107) = 1.98 | 0.143 | 0.018 | — |
| ABAS-3 Practical | 91.0 [85.2, 96.8] | 86.7 [82.1, 91.3] | 84.7 [79.9, 89.5] | *F*(2,107) = 1.39 | 0.254 | 0.007 | — |
| Self-rated MASC-2/CDI-2 (T-score) |  |  |  |  |  |  |  |
| MASC-2 Total | 45.7 [41.5, 49.9] | 65.0 [61.7, 68.3] | 60.7 [57.4, 64.1] | *F*(2,112) = 26.87 | < 0.001* | 0.310 | MH-P_1_N_1_, MH-P_0_N_1_ > MH-P_0_N_0_ |
| CDI-2 Total | 46.0 [42.0, 50.1] | 60.8 [57.7, 63.9] | 64.7 [61.4, 68.0] | *F*(2,113) = 26.87 | < 0.001* | 0.308 | MH-P_1_N_1_, MH-P_0_N_1_ > MH-P_0_N_0_ |
| Self-rated Conners-3 (T-score) |  |  |  |  |  |  |  |
| Inattention | 53.7 [46.8, 60.5] | 70.3 [64.2, 76.4] | 72.4 [66.3, 78.5] | *F*(2,50) = 9.76 | < 0.001* | 0.249 | MH-P_1_N_1_, MH-P_0_N_1_ > MH-P_0_N_0_ |
| Hyperactivity/Impulsivity | 50.2 [42.9, 57.5] | 64.9 [58.4, 71.4] | 65.4 [58.9, 71.9] | *F*(2,50) = 6.05 | 0.004* | 0.160 | MH-P_1_N_1_, MH-P_0_N_1_ > MH-P_0_N_0_ |
| Learning Problems | 55.0 [48.4, 61.6] | 71.3 [65.4, 77.2] | 68.6 [62.7, 74.5] | *F*(2,50) = 7.52 | 0.001* | 0.198 | MH-P_1_N_1_, MH-P_0_N_1_ > MH-P_0_N_0_ |
| Defiance/Aggression | 47.1 [39.5, 54.7] | 54.6 [47.8, 61.3] | 63.6 [56.9, 70.4] | *F*(2,50) = 5.43 | 0.007* | 0.143 | MH-P_1_N_1_ > MH-P_0_N_0_ |
| Family Problems | 45.2 [38.6, 51.8] | 53.3 [47.4, 59.1] | 56.4 [50.6, 62.3] | *F*(2,50) = 3.41 | 0.041* | 0.083 | MH-P_1_N_1_ > MH-P_0_N_0_ |
| Parent-rated Conners-3 (T-score) |  |  |  |  |  |  |  |
| Inattention | 64.8 [59.0, 70.6] | 70.4 [66.0, 74.8] | 76.9 [72.0, 81.7] | *F*(2,114) = 5.18 | 0.007* | 0.067 | MH-P_1_N_1_ > MH-P_0_N_0_ |
| Hyperactivity/Impulsivity | 60.8 [54.4, 67.2] | 66.4 [61.6, 71.2] | 68.9 [63.6, 74.2] | *F*(2,114) = 1.91 | 0.153 | 0.015 | — |
| Learning Problems | 69.5 [63.6, 75.4] | 71.1 [66.6, 75.5] | 70.6 [65.6, 75.5] | *F*(2,114) = 0.08 | 0.920 | -0.016 | — |
| Executive Functioning | 58.6 [52.9, 64.3] | 67.8 [63.6, 72.0] | 71.7 [67.0, 76.4] | *F*(2,109) = 6.29 | 0.003* | 0.086 | — |
| Defiance/Aggression | 51.4 [45.3, 57.6] | 60.9 [56.2, 65.5] | 67.0 [61.9, 72.2] | *F*(2,114) = 7.41 | 0.001* | 0.099 | MH-P_1_N_1_, MH-P_0_N_1_ > MH-P_0_N_0_ |
| Peer Relations | 57.3 [50.6, 64.0] | 63.5 [58.4, 68.6] | 66.7 [61.1, 72.3] | *F*(2,114) = 2.30 | 0.105 | 0.022 | — |

*Note.* Pairwise contrasts (Tukey HSD) significant at the *p* < 0.05 level are displayed in the “Significant Contrasts” column. MH-P_0_N_0_ = no mental health diagnoses from neuropsychologist; MH-P_0_N_1_ = no mental health concerns on intake interview, but diagnosed with mental health disorder by neuropsychologist; MH-P_1_N_1_ = mental health concerns raised by parent on intake interview and confirmed by neuropsychologist; EMM = estimated marginal (least squares) mean; ABAS-3 = Adaptive Behavior Assessment System–3; GAC = General Adaptive Composite; MASC-2 = Multidimensional Anxiety Scale for Children–2; CDI-2 = Children's Depression Inventory–2.

* *p* < 0.05
