# Supplementary material for: Parent and Provider Differences in Ratings of Mental Health and Neurodevelopmental Concerns in Children with Neurologic Disorders
Source: J Clin Psychol Med Settings. Author manuscript; Available in PMC 2024 Sep 1. (PMC11333177; doi:10.1007/s10880-023-09990-0)
Supplement: Supplementary file 2 [file NIHMS1970392-supplement-Supplemental_Table_2.docx]

**Supplemental Table 2.**

***Estimated Marginal Means and Comparisons Among Neurodevelopmental Concern Groups***

| **Outcome** | **ND-P**_**0**_**N**_**0**_ **Group** **(EMM [95% CI])** | **ND-P**_**0**_**N**_**1**_ **Group** **(EMM [95% CI])** | **ND-P**_**1**_**N**_**1**_ **Group** **(EMM [95% CI])** | **Omnibus Test** | ***p*** | **ω**^**2**^ | **Significant Contrasts** |
| --- | --- | --- | --- | --- | --- | --- | --- |
| Adaptive Behavior (Standard Score) |  |  |  |  |  |  |  |
| ABAS-3 GAC | 93.7 [89.1, 98.2] | 90.5 [86.1, 95.0] | 75.5 [71.5, 79.4] | *F*(2,108) = 21.71 | < 0.001* | 0.272 | ND-P_1_N_1_ < ND-P_0_N_1_, ND-P_0_N_0_ |
| ABAS-3 Conceptual | 93.7 [88.5, 98.8] | 87.2 [82.3, 92.2] | 77.0 [72.6, 81.5] | *F*(2,110) = 12.11 | < 0.001* | 0.164 | ND-P_1_N_1_ < ND-P_0_N_1_, ND-P_0_N_0_ |
| ABAS-3 Social | 96.0 [91.2, 100.8] | 92.4 [87.6, 97.2] | 78.7 [74.4, 82.9] | *F*(2,107) = 16.54 | < 0.001* | 0.220 | ND-P_1_N_1_ < ND-P_0_N_1_, ND-P_0_N_0_ |
| ABAS-3 Practical | 95.1 [90.6, 99.7] | 92.2 [87.9, 96.6] | 76.8 [72.8, 80.7] | *F*(2,107) = 22.21 | < 0.001* | 0.273 | ND-P_1_N_1_ < ND-P_0_N_1_, ND-P_0_N_0_ |
| Self-rated MASC-2/CDI-2 (T-score) |  |  |  |  |  |  |  |
| MASC-2 Total | 58.5 [54.2, 62.8] | 61.2 [56.9, 65.5] | 56.5 [52.2, 60.7] | *F*(2,112) = 1.22 | 0.299 | 0.004 | — |
| CDI-2 Total | 57.2 [53.0, 61.3] | 60.4 [56.3, 64.5] | 58.4 [54.2, 62.5] | *F*(2,113) = 0.62 | 0.541 | -0.007 | — |
| Self-rated Conners-3 (T-score) |  |  |  |  |  |  |  |
| Inattention | 56.3 [48.6, 63.9] | 68.8 [62.4, 75.3] | 71.2 [64.6, 77.7] | *F*(2,50) = 4.88 | 0.012* | 0.128 | ND-P_1_N_1_, ND-P_0_N_1_ > ND-P_0_N_0_ |
| Hyperactivity/Impulsivity | 49.1 [43.0, 55.2] | 56.3 [51.2, 61.4] | 74.5 [69.2, 79.7] | *F*(2,50) = 22.87 | < 0.001* | 0.452 | ND-P_1_N1 > ND-P_0_N_1_, ND-P_0_N_0_ |
| Learning Problems | 58.6 [51.1, 66.0] | 68.5 [62.3, 74.7] | 68.0 [61.6, 74.4] | *F*(2,50) = 2.50 | 0.123 | 0.044 | — |
| Defiance/Aggression | 45.7 [39.4, 52.0] | 49.0 [43.7, 54.3] | 70.1 [64.7, 75.5] | *F*(2,50) = 22.75 | < 0.001* | 0.451 | ND-P_1_N1 > ND-P_0_N_1_, ND-P_0_N_0_ |
| Family Problems | 50.1 [42.9, 57.3] | 51.9 [45.8, 57.9] | 53.9 [47.7, 60.1] | *F*(2,50) = 0.33 | 0.718 | -0.026 | — |
| Parent-rated Conners-3 (T-score) |  |  |  |  |  |  |  |
| Inattention | 59.7 [55.2, 64.2] | 70.7 [66.5, 74.9] | 81.4 [88.3, 85.5] | *F*(2,114) = 24.94 | < 0.001* | 0.290 | ND-P_1_N_1_ > ND-P_0_N_1_ > ND-P_0_N_0_ |
| Hyperactivity/Impulsivity | 52.8 [48.0, 57.6] | 66.3 [61.8, 70.8] | 76.5 [72.1, 80.8] | *F*(2,114) = 25.98 | < 0.001* | 0.299 | ND-P_1_N_1_ > ND-P_0_N_1_ > ND-P_0_N_0_ |
| Learning Problems | 63.1 [58.2, 68.0] | 69.5 [65.0, 74.1] | 77.6 [73.2, 82.1] | *F*(2,114) = 9.60 | < 0.001* | 0.128 | ND-P_1_N_1_ > ND-P_0_N_1_, ND-P_0_N_0_ |
| Executive Functioning | 55.5 [51.2, 59.8] | 66.8 [62.7, 70.8] | 76.9 [72.9, 80.9] | *F*(2,109) = 26.08 | < 0.001* | 0.309 | ND-P_1_N_1_ > ND-P_0_N_1_ > ND-P_0_N_0_ |
| Defiance/Aggression | 49.9 [44.9, 55.0] | 58.8 [54.1, 63.5] | 71.5 [66.9, 76.1] | *F*(2,114) = 20.22 | < 0.001* | 0.247 | ND-P_1_N_1_ > ND-P_0_N_1_ > ND-P_0_N_0_ |
| Peer Relations | 56.4 [50.7, 62.2] | 60.6 [55.3, 66.0] | 71.0 [65.8, 76.3] | *F*(2,114) = 7.55 | 0.001* | 0.101 | ND-P_1_N_1_ > ND-P_0_N_1_, ND-P_0_N_0_ |

*Note.* Pairwise contrasts (Tukey HSD) significant at the *p* < 0.05 level are displayed in the “Significant Contrasts” column. ND-P_0_N_0_ = no neurodevelopmental diagnoses from neuropsychologist; ND-P_0_N_1_ = no neurodevelopmental concerns on intake interview, but diagnosed with neurodevelopmental disorder by neuropsychologist; ND-P_1_N_1_ = neurodevelopmental concerns raised by parent on intake interview and confirmed by neuropsychologist; EMM = estimated marginal (least squares) mean; ABAS-3 = Adaptive Behavior Assessment System–3; GAC = General Adaptive Composite; MASC-2 = Multidimensional Anxiety Scale for Children–2; CDI-2 = Children's Depression Inventory–2.

* *p* < 0.05
